# Effect of Temperature, Added Calcium and pH on the Equilibrium of Caseins between Micellar State and Milk Serum

**DOI:** 10.3390/foods10040822

**Published:** 2021-04-10

**Authors:** Simon Schiffer, Eva Scheidler, Tim Kiefer, Ulrich Kulozik

**Affiliations:** Chair of Food and Bioprocess Engineering, TUM School of Life Sciences, Technical University of Munich, 85354 Freising, Germany; eva.scheidler@tum.de (E.S.); tim.fabian.kiefer@hs-furtwangen.de (T.K.); ulrich.kulozik@tum.de (U.K.)

**Keywords:** casein micelle, serum casein, colloidal interactions

## Abstract

Micellar casein and casein monomers in milk serum are in a dynamic equilibrium. At temperature below 15–20 °C a considerable amount of casein monomers, β-casein in particular, is released from the casein micelle into the aqueous serum phase. This study investigates the effects of added calcium and related variations of pH on this peculiar equilibrium in order to minimize the amount of caseins in the serum and to better understand the casein permeation during microfiltration. The pH was varied in the range of 6.3 to 7.3 and the content of calcium was increased up to 7.5 mM by adding CaCl_2_. Upon equilibration, the milk was separated by ultracentrifugation and the amounts of protein in the supernatant were analyzed. It was shown that the addition of low amounts of calcium shifts the equilibrium towards the micellar casein phase and can, thus, lower the serum casein content induced at low temperatures. Relative to that, the adjustment of pH separately from the CaCl_2_ addition had a minor effect on casein concentration and composition in the serum.

## 1. Introduction

Caseins in milk exist in the form of micelles and to a smaller extent as soluble monomers in the milk serum. These two fractions stay in a dynamic equilibrium, depending on various factors, e.g., temperature and ionic environment [1]. The structural interaction of the four main casein monomers, namely α_S1_, α_S2_-, β-, and κ-casein, forming micelles is described either by the calcium-phosphate nanocluster model [2,3] or by the dual binding model [4]. Hereby, the calcium content in particular is of relevance as a stabilizing factor of the micellar structure [5]. Temperatures below 15 °C lead to an increased release of casein monomers from the casein micelles into the aqueous serum [6,7,8]. The equilibrium between soluble and micellar bound casein can be modified on purpose or unintentionally as a consequence of processing and compositional variations.

One of these unintended side effects is an increased serum casein concentration affecting milk protein fractionation by means of microfiltration (MF), if conducted at low temperatures, as reported, e.g., Schiffer et al. [9] or France et al. [10], instead of 50–55 °C [11,12]. This can negatively affect the fractionation result by an increased transfer of casein monomers together with the equally sized whey proteins into the MF permeate, thus reducing the purity of the whey protein fraction. The whey protein fraction is the target fraction meant to be obtained in the permeate, except for some specific applications, to obtain β-casein enriched MF permeate [13]. Lower temperatures also increase the solubility of calcium and phosphate [14] and the pH of milk [15]. The solubility of α_S_- and β-casein also depends on pH and, thus, indirectly on temperature [16].

**Figure 1:**
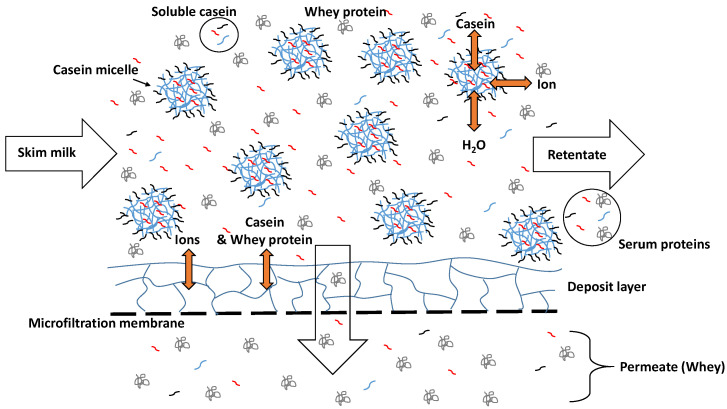
Schematic representation of the separation of casein and whey protein regarding a skim milk microfiltration.

To reduce the effect of the release of casein monomers into the serum at low temperatures, Schiffer et al. [17] studied the effect of calcium chloride addition in low amounts to skim milk prior to microfiltration. The authors reported that this in fact reduces the permeation of casein monomers into the MF permeate, which could be seen as the equivalent to the milk serum. However, this effect could not be clearly attributed to changes in the dynamic casein micelle/serum casein equilibrium as such, because the calcium addition also induces a tighter deposited casein layer structure of the retained casein micelles on the membrane surface. Therefore, a reduction of casein monomer concentration in the MF permeate could also be attributed to the increased retention capacity of the deposited casein layer instead of a reduction of casein monomers in the milk serum (Figure 1).

Even though the serum casein reducing effect of a calcium addition to milk was shown [18] for selected temperature conditions, a systematic and wider investigation across the entire relevant 6–20 °C range, which is of particular interest regarding MF process optimization, is lacking. Another aspect is that the addition of CaCl_2_ to milk also results in a pH reduction [19]. Besides the enhancement of ionic interactions between the caseins upon calcium addition, pH related electrostatic effects could occur, thus changing protein–protein interactions.

Therefore, to better understand the effects of a calcium enrichment on the equilibrium between micellar bound and soluble casein at low temperature, the milk serum can be separated by ultracentrifugation [8,20] without the retention effects of the conditions prevailing in microfiltration.

It was the aim of this study to better understand the complexity of combined effects between temperature, pH, and calcium addition on the equilibrium between micellar bound and soluble caseins as well as between ions. The purpose was to gain new insights into protein–protein interactions between casein species as a function of aqueous phase composition and temperature-induced changes in the casein micelle. The temperature was varied in a range of 6 and 20 °C to induce the release of mainly β-casein into the serum at various levels. To assess the impact of the pH resulting from the temperature shift, different pH values were moderately adjusted at the respective temperatures in a range between pH 6.3 and 7.3. Furthermore, 0–7.5 mM calcium were added (in the form of CaCl_2_) to investigate its effect on the serum casein concentration. To separate the structuring effect of calcium from the altered electrostatic repulsion resulting from the CaCl_2_ induced change in pH, an additional pH re-adjustment after the addition of different amounts of CaCl_2_ was performed.

## 2. Materials and Methods

### 2.1. Skim Milk and Preparation for Experiments

Pasteurized skim milk (74 °C/28 s) was obtained from the local dairy (Molkerei Weihenstephan GmbH & Co. KG, Freising, Germany). For experiments with an enhanced calcium concentration, a 1 M CaCl_2_ solution was added in different amounts and the milk was stirred overnight at 4 °C before use. Before, an experiment was conducted the milk was stored for at least 12 h at 4 °C. The samples were held at the target temperature (6–20 °C) for 60 min to achieve the new equilibrium between soluble and micellar bound casein, following a report by Liu et al. [20]. Then, the pH was adjusted with 1 M potassium hydroxide solution and 1 M hydrochloric acid and the samples were tempered for an additional 15 min. This resulted in a temperature profile where the samples were warmed up from 4 °C to 6–20 °C and tempered for 60 min. Then, the pH was adjusted at the particular temperature and after additional 15 min the samples were immediately placed into the tempered ultracentrifuge.

### 2.2. Separation of Micellar and Soluble Casein

**Figure A1:**
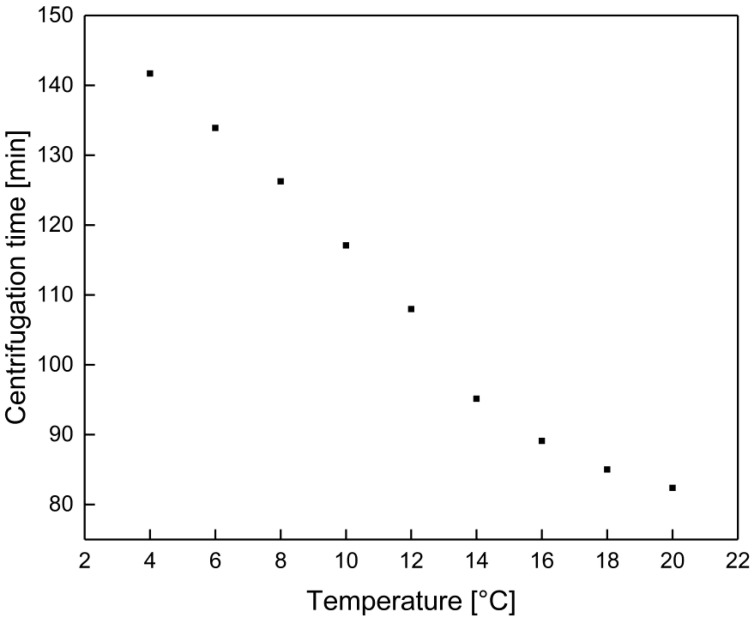
Centrifugation time as a function of the temperature.

Ultracentrifugation was conducted with an Ultracentrifuge Sorvall WX80 (Thermo Fisher Scientific, Waltham, MA, USA). The ultracentrifuge and the rotor head (Fiberlite F50L-24x1.5 Rotor, Thermo Fisher Scientific, Waltham, MA, USA) were tempered to the samples’ respective temperatures. 1 mL of sample was filled into a 1.5 mL Ultra Microtube (Thermo Fisher Scientific, Waltham, MA, USA). The required centrifugation time at 72,000× *g* was adjusted to the temperature related viscosity (measured by a MCR 302 rheometer from Anton Paar GmbH, Graz, Austria), this centrifugation force is sufficient to separate the soluble milk proteins from micellar or aggregated casein structures as shown by Dumpler et al. [21]. A shift in the temperature changes the milk viscosity and therefore, the conditions required to obtain a constant separation effect. To exclude a shift in the separation efficiency, due to a variation in the centrifugal force [22] the process time was adjusted as a function of the temperature. The centrifugation time was calculated with the dynamic viscosity of the serum phase (*η*), the density of the protein (*σ*) and serum phase (δ), the critical diameter of proteins which should precipitated (*d*), the height of the filling level of the samples (*x*), the radius of the samples in the rotor head (*R*), as well as the rotation speed (*n*). The calculated centrifugal time as a function of the temperature is shown in the Appendix A (Figure A1).

(1) $$t_{s}=\frac{18\eta }{\left(\sigma -\delta \right)*d^{2}}*\frac{x}{R\;*{\left(2*\pi *n\right)}^{2}}.$$

### 2.3. Analyses

**Figure A2:**
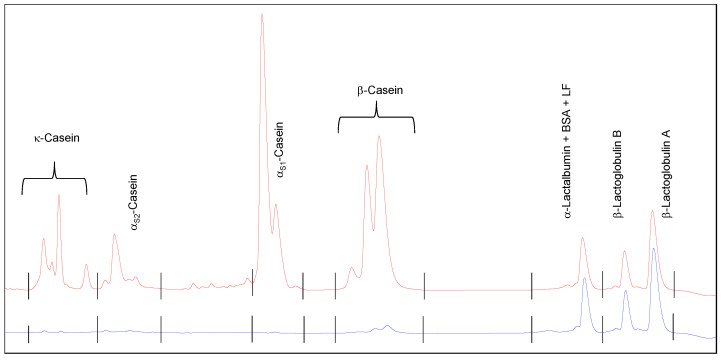
Chromatogram of the protein composition in skim milk (**red line**) as well as in the serum phase after ultracentrifugation (**blue line**).

The quantification of the proteins was conducted via reversed phase high performance liquid chromatography (RP-HPLC) using an Agilent 1100 Series chromatograph (Agilent Technologies, Waldbronn, Germany) with a C18 analytical silica-based column (Agilent Zorbax 300SB-C18, 4.6 × 150 mm, 5 μm). The simultaneous quantitative analysis of the whey protein and casein fractions (α_S1_-, α_S2_-, β-, and κ-casein) was performed applying a method developed by Dumpler et al. [21], via a dilution of the samples in a guanidine puffer. During the analysis, solvent A (0.1% trifluoroacetic acid (TFA) in 90% HPLC grade water and 10% acetonitrile) as well as solvent B (0.07% TFA in 10% HPLC grade water and 90% acetonitrile) were used with a flow rate of 1.2 mL min^−1^ at 40 °C. A chromatogram of the protein profile of skim milk as well as the serum phase after ultracentrifugation is shown in the Appendix A (Figure A2). The total casein concentration was calculated by adding up the respective casein fractions of α_S1_-, α_S2_-, β-, and κ-casein.

For the determination of the ion concentration in the serum phase after ultracentrifugation, the protein fraction was removed. The serum phase was diluted 1:4 with Milli-Q-water and filtered by a modified PES 10 K filter (VRW, Radnor, PA, USA) under centrifugal conditions at 10,000× *g* for 10 min (Z 233 M-2, Hermle Labortechnik GmbH, Wehingen, Germany). To minimize the loss of ions remaining in the filter unit, the filters were flushed twice with 500 µL MilliQ-water under the same centrifugation conditions at 10,000× *g* for 10 min. Quantification of the ion concentration in the serum phase was carried out according to the method described by Dumpler et al. [23].

### 2.4. Data Evaluation and Statistics

Data plotting and statistical data evaluation was performed using OriginPro 2017G (OriginLab Corporation, Northampton, MA, USA). The statistical analysis was performed using data series as a function of temperature (8 settings), pH (5 settings), calcium addition (5 settings) as well as calcium addition with pH adjustment (6 settings). All setting combinations were done in duplicates. The standard deviation from the mean value was calculated within a data series. Statistical significance between or within a data series was evaluated using analysis of variance (ANOVA) as well as a *t*-test. The calculated *p*-values are given in the text and indicate the significance level (*p* ≤ 0.05).

## 3. Results and Discussion

### 3.1. Serum Concentration of β-Casein Lowers with Increasing Temperature

**Figure 2:**
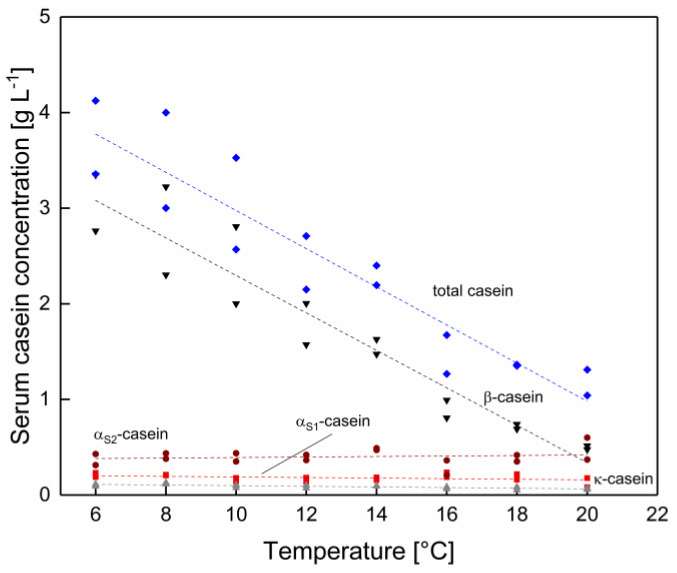
Protein concentration in the serum phase as a function of temperature in Δϑ steps of 2 °C for total casein concentration (◆) and the casein species α_S1_- (■), α_S2_- (●), β- (▼), and κ-casein (▲) (*n* = 2). Data points are individual experiments. The lines are a guide to the eye. Total casein and β-casein concentration change significantly (*p* < 0.05) and α_S1_-, α_S2_ and κ-casein concentration not significant (*p* > 0.05) as a function of the temperature.

At first, the effect of temperature as such was assessed. As shown in Figure 2, the total serum casein concentration decreases linearly with increasing temperature. β-casein was identified as the casein monomer with the most pronounced changes in the serum concentration. This could be due to its primarily hydrophobic protein–protein interactions [7]. In contrast to β-casein, the concentrations of α_S1_-, α_S2_- and κ-casein stayed nearly constant. The ionic stabilizing effects determining the integration of α_S1-_ and α_S2_-caseins in the micelle are likely to be only slightly affected by a temperature shift [24]. The constant κ-casein concentration is assumed to occur, due to the fact that κ-casein shows low calcium phosphate binding capacity, glycosylation patterns enable hydrophilic protein–protein interactions [25]. As a result, the pronounced effect of temperature on the serum β-casein concentration changes the casein monomer ratio of the serum phase as a function of the temperature.

Temperature not only affects the equilibrium of micellar and serum caseins, but also the concentration of soluble ions in the serum phase. With increasing temperature, the pH of milk decreased slightly from 6.78 at 6 °C to 6.72 at 20 °C (Table A1 in Appendix A). However, the mean anion and cation concentration in the serum phase did not change significantly in the temperature range between 6–20 °C, as shown in Table A2 and Table A3 in the Appendix A (*p* > 0.05). As reported by Dumpler et al. [23] a shift in the ion equilibria can occur if the temperature is further increased up to 50 °C.

### 3.2. Effect of pH on the Serum Casein Concentration

As shown in Section 3.1 with decreasing temperature the casein concentration in the serum phase as well as the pH increased. To observe whether the temperature induced changes in the pH have an additional effect on the casein equilibrium, the pH was adjusted for each temperature between 6–20 °C in 2 °C intervals to pH 6.3, 6.5, 7.0, and 7.3. This moderate changes were applied to determine if not only changes in the hydrophobic interactions but also the shift in the pH, has an effect on the solubility of the single casein species.

**Figure 3:**
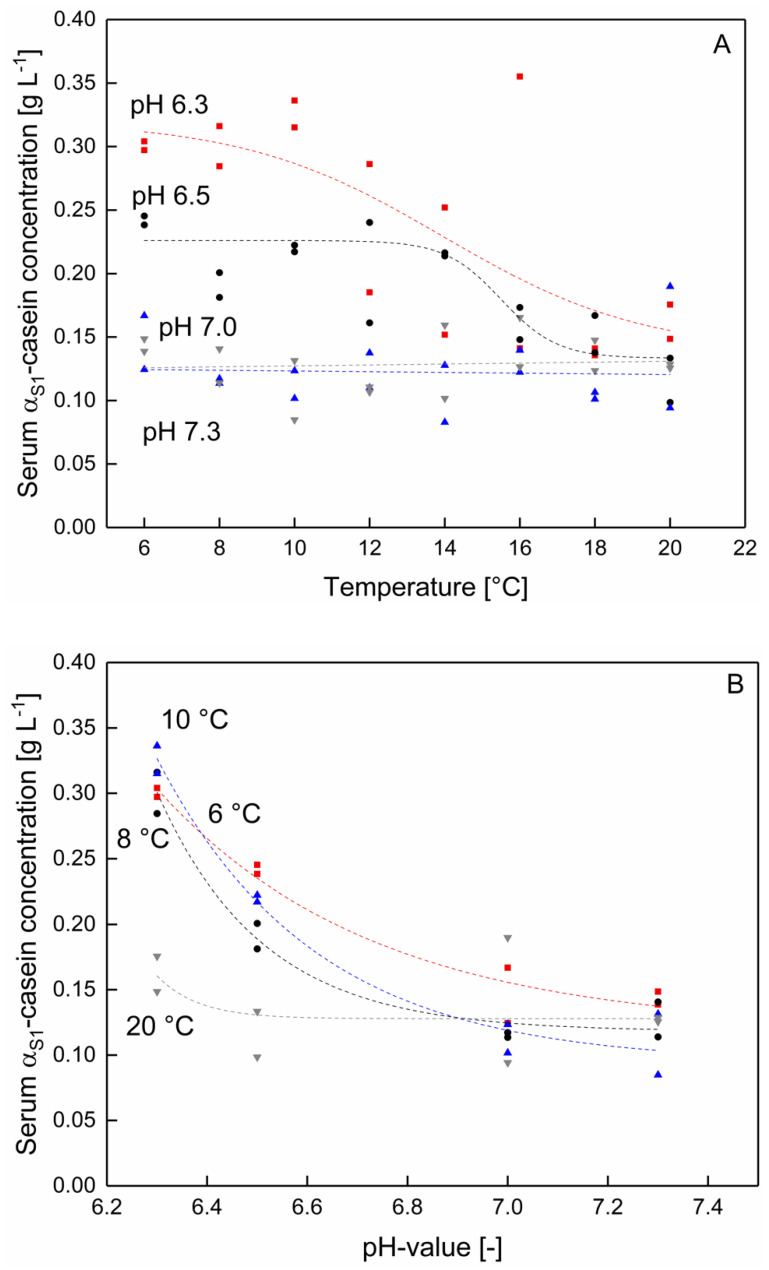
α_S1_-casein concentration in the serum phase (**A**) as a function of the temperature at pH 6.3 (■), 6.5 (●), 7.0 (▲), and 7.3 (▼) and (**B**) as a function of the pH at 6 (■), 8 (●), 10 (▲), and 20 °C (▼) (*n* = 2). Data points are individual experiments. The lines are a guide to the eye. α_S1_-concentration changes significantly between 6–10 °C as function of the pH (7.0–6.3) (*p* < 0.05). Changes between pH 7.0 and 7.3 at 6–20 °C and between pH 6.0–7.3 at 12–20 °C were not significant (*p* > 0.05).

**Figure 4:**
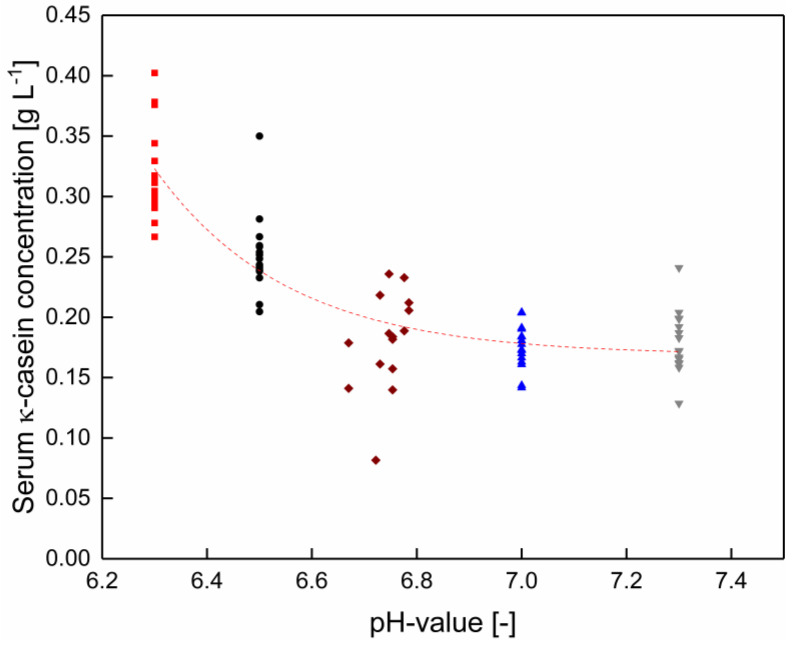
κ-casein concentration in the serum phase without pH adjustment (◆), as well as at pH 6.3 (■), 6.5 (●), 7.0 (▲), and 7.3 (▼) between 6–20 °C for each pH (*n* = 2). Data points are individual experiments. The red line is a guide to the eye. κ-casein concentration changes significantly between the skim milk without and with a pH adjustment to 6.0 (*p* < 0.05).

It could be shown that serum κ-casein and α_S1_-casein concentration significantly changed as a function of pH (at temperatures between 6–20) (Figure 3 and Figure 4) (*p* < 0.05). In contrast to that, the serum concentration of β‑casein as well as α_S2_‑casein were not significantly affected by the pH (data not shown) (*p* > 0.05). Therefore, in this section only data for κ-casein and α_S1_-casein are presented.

As shown in Figure 3A, the serum concentration of α_S1_-casein increases with decreasing pH in the range of 6.5–6.3, whereas after an increase towards pH 7.0 and 7.3, the α_S1_-casein concentration remained unaffected. As can be seen the pH affects the α_S1_ serum casein concentration mainly in the low temperature range between 6–10 °C (Figure 3B). The temperature dependent solubility of α_S1_-caseins is in accordance with the findings of Post et al. [16]. The authors reported a solubility maximum of α_S_-casein in micellar casein solutions at 2 °C at approx. pH 5.5–5.2, which could not be observed at 20 °C. Therefore, with the data shown in Figure 3B it can be specified that a shift in the solubility of α_S1_ occurs as a function of the pH at temperatures as low as 10 °C. It is assumed that at lower temperatures both binding mechanisms of α_S1_‑caseins within the casein micelle, hydrophobic interactions as well as the binding to calcium phosphate clusters [25] are weakened and thus the solubility is increased.

The κ-casein serum concentration decreased with increasing pH. In contrast to α_S1_-casein, this effect was independent of the temperature (Figure 4). At pH values between native conditions and pH 7.3, the κ-casein concentration is constant (*p* > 0.05). κ-casein is the weakness calcium phosphate binding protein of all casein species [26]. The increasing κ-casein concentration in the serum phase with decreasing pH is linked to an increasing calcium solubility and therefore, reduced interactions between caseins and ions as well as proteins in the micellar structure [24,27].

### 3.3. Increasing Calcium and Phosphate Concentration in the Serum Phase with Decreasing pH

A decrease of the pH leads to a mutual effect of an H^+^ enrichment as well as a shift in the ion equilibrium. Therefore, it was assumed in Section 3.2 that changes in the ion equilibrium as a function of the pH contribute to the observed pH-dependent changes in the serum concentration of α_S1_- and κ-casein. As shown for phosphate and for calcium in Table 1, the serum concentrations of these ions were significantly affected by the pH (*p* < 0.05). However, at pH 7.0 and 7.3 the calcium concentration does not change significantly (*p* > 0.05), comparable to the behavior of α_S1_- and κ-casein. By lowering the pH micellar ions dissociate into the serum [28]. An increase of the citrate concentration could be observed as well when the pH was decreased to 6.3, independently of the temperature (*p* > 0.05) (Table 1) The magnesium concentration neither changed significantly with the temperature, nor with the pH within the pH range studied (*p* > 0.05) (Table 1).

**Figure A3:**
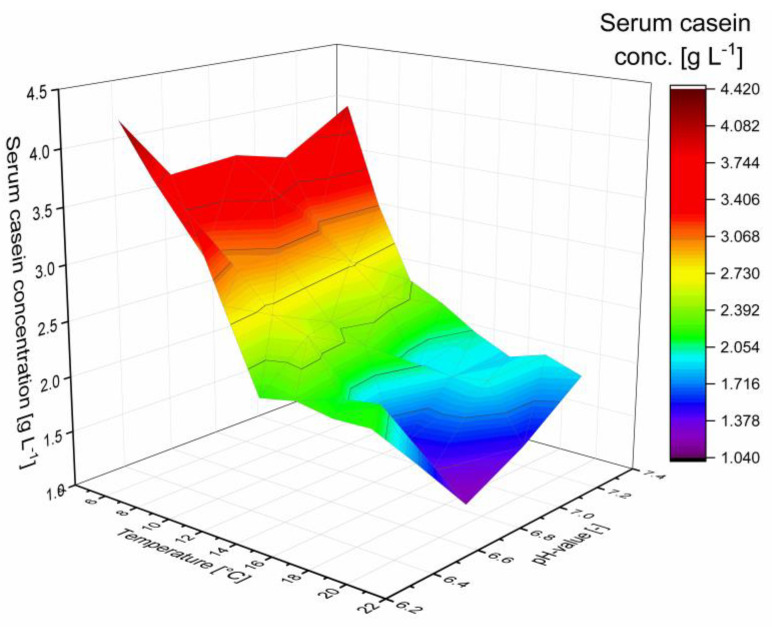
Total serum casein concentration at 6–20 °C as a function of the pH (6.3–7.3).

In conclusion, it could be shown that changes in the total serum casein concentration are affected by a temperature variation, whereas the pH did not have a significant effect on the casein micelle/serum casein equilibrium (data shown in Figure A3 in the Appendix A) (*p* > 0.05). Even though the total serum casein concentration in this temperature range cannot be influenced by changing the pH in a moderate range, it is possible to affect the single casein species α_S1_- and κ-casein as well as the ion equilibrium.

### 3.4. Decrease of the Serum Casein Concentration with Increasing Calcium Enrichemt

To decrease the serum casein concentration, calcium was enriched in order to bind within calcium phosphate clusters to the caseins, strengthening the casein–casein interaction and forming casein aggregates.

**Figure 5:**
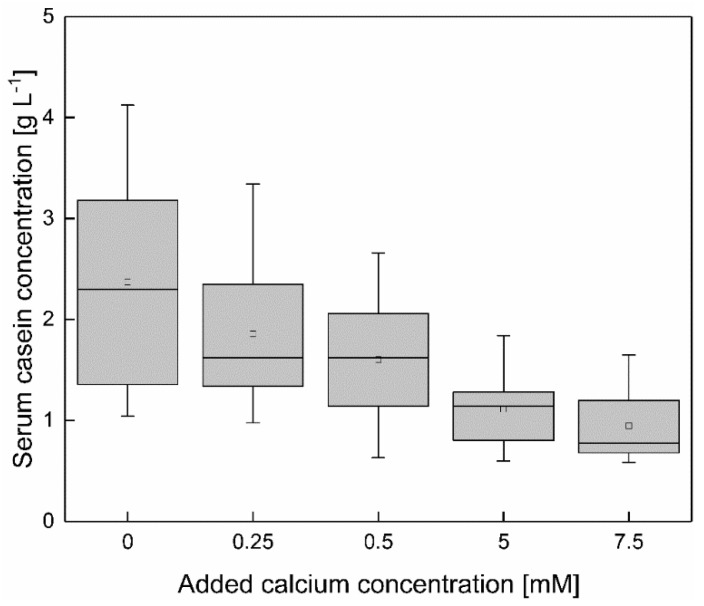
Mean value of the total serum casein concentration of skim milk between 6–20 °C at different levels of calcium addition, shown in a box plot for all casein concentrations with the same levels of added calcium. Serum casein concentration (between 6–20 °C) decreased significantly with increasing calcium addition (*p* < 0.05).

**Figure A4:**
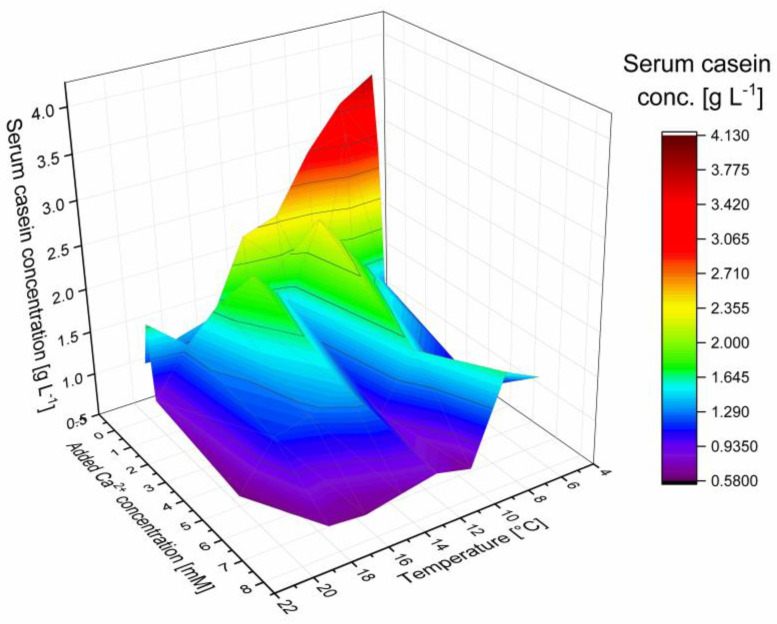
Total serum casein concentration at 6–20 °C as a function of added calcium (0–7.5 mM).

To determine the effect of a calcium addition the mean serum casein concentration over the total temperature range from 6–20 °C was compared as a function of the calcium enrichment (Figure 5). In Section 3.1 it could be shown that the serum casein concentration is linearly decreasing with increasing temperature. This linear trend as a function of temperature can also be observed with increasing calcium concentration (shown in Appendix A Figure A4). It can be determined that the temperature dependency of the serum casein content in the range of 6–20 °C decreases with increasing amount of added calcium until it vanishes completely at a concentration of 0.50 mM added calcium.

The casein concentration as a function of the temperature shows a linear dependency at 0–7.5 mM calcium. Therefore, the effect of a calcium enrichment is shown via the comparison of the mean casein concentration, between 6–20 °C (*n* = 2), at 0–7.5 mM added calcium. As shown in Figure 5, increasing the calcium concentration (ϑ = 6–20 °C) reduces the serum casein concentration almost steadily. To determine significant changes in the serum casein concentration as a function of a calcium enrichment the results as shown in Figure 5 (mean casein concentration between 6–20 °C at 0–7.5 mM added calcium) were compared via *t*-test. Between the casein concentrations as a function of temperature of skim milk with 0.00 and 0.25 mM added calcium, no significant difference could be observed (*p* > 0.05). Therefore, the enrichment with 0.25 mM calcium was not sufficient to affect the casein equilibrium. A further increase of the added calcium concentration to 0.5–7.5 mM showed a significant reduction of the serum casein concentration, compared to native conditions (*p* < 0.05). However, an enhancement of the calcium concentration from 5–7.5 mM calcium did not result in a further decrease in serum casein concentration.

Even though increasing the calcium addition beyond 0.50 mM had no effect on the temperature dependency of the serum casein concentration, as described above, the total serum casein concentration can be decreased up to 5.00 mM. The conclusion is that 0.50 mM calcium are required to overcome the temperature dependency of the serum casein content but a reducing effect on the serum casein concentration can be observed up to an addition of 5.00 mM calcium.

As reported by Holt et al. [24] and Horne [29], the calcium sensitivity of the different casein species is dependent on the concentration of phosphorylated amino acids in the primary structure, which increases from α_S2_ > α_S1_ > β > κ. However, all casein species are affected significantly by the addition of calcium chloride (*p* < 0.05). In the following, it should be kept in mind that besides the enhancement of ionic interactions between calcium and serum caseins, the addition of CaCl_2_ also lowers the pH [19]. This could have an unintended side effect influencing the micelle/serum casein equilibrium comparable to the results in Section 3.2.

**Figure 6:**
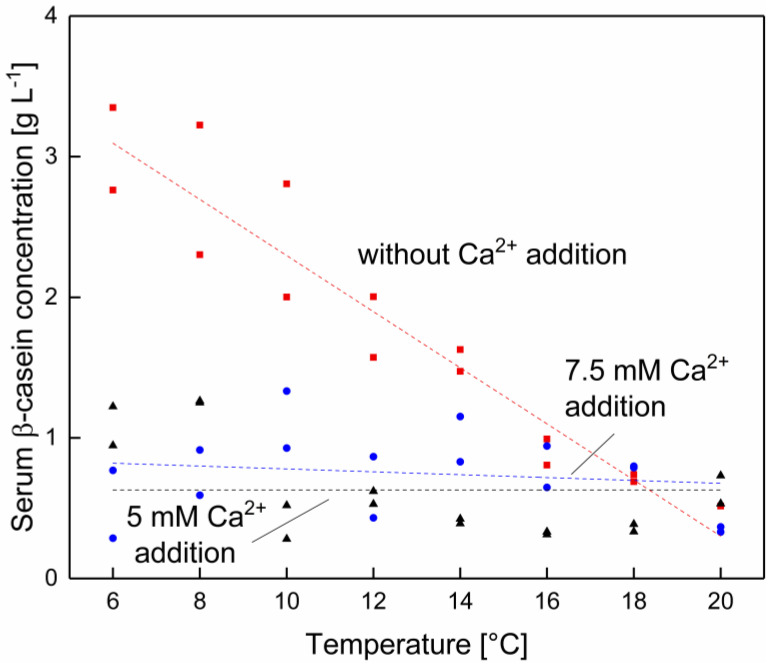
β-casein concentration in the serum phase of skim milk at different levels of calcium addition at 6–20 °C with 0 (■), 5 (●), and 7.5 mM (▲) of added calcium (*n* = 2). Data points are individual experiments. The lines are a guide to the eye. Without calcium addition a significant (*p* < 0.05) and with 5–7.5 mM added calcium no significant (*p* > 0.05) change of the β-casein concentration as a function of the temperature was determined.

As can be seen in Figure 6, it is possible to decrease the β-casein concentration as a result of a calcium addition. At lower temperatures, the β-casein serum concentration can be reduced by adding calcium, but this effect vanishes as the temperature approaches 20 °C. Between 16–20 °C there seems to be no reduction in the serum β-casein concentration by increasing the calcium enrichment. Furthermore, after the addition of 5 mM calcium, there seems to be that the lowest possible serum β-casein level of 0.6 g L^−1^ across whole temperature range is reached, which is similar to the β-casein concentration in native skim milk at 20 °C. It is therefore assumed that it is possible to decrease the serum β-casein concentration at low temperatures and to counterbalance the decreased hydrophobic interactions, which result in an increased dissociation of β-casein from micellar structures. Effects of calcium addition at low temperature on the casein equilibrium are likely due to changes in the zeta potential, hydrophobicity and ionic interactions, decreasing the serum concentration as shown in Figure 6 [30,31]. Philippe et al. [30] could observe at room temperature a reduction of the β-casein concentration upon addition of 4.5 mM calcium. However, as depicted in Figure 6, the already low amount of β-casein determined at 16–20 °C could not be further reduced by the addition of calcium.

**Figure 7:**
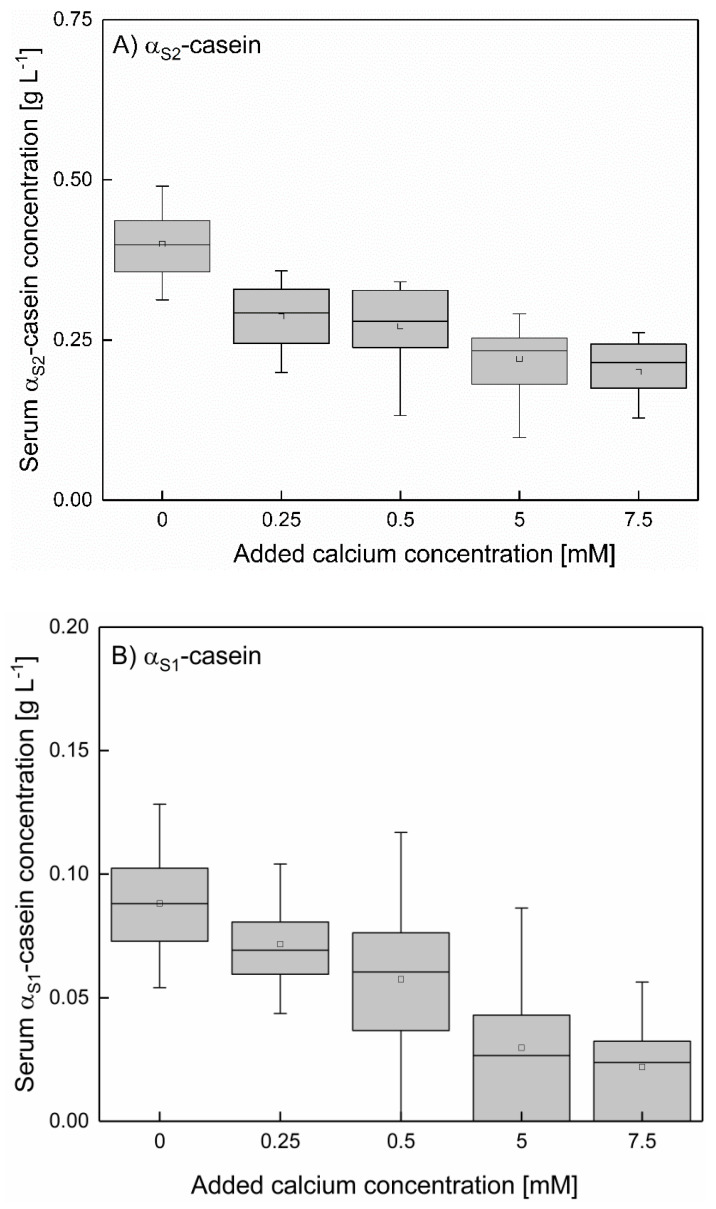
Mean value of the serum α_S2_- (**A**) and α_S1_-casein (**B**) of skim milk between 6–20 °C at different levels of calcium addition, shown in a box plot for all casein concentrations with the same levels of added calcium. α_S_–casein concentration (mean value between 6–20 °C) decreased significantly with increasing calcium addition (*p* < 0.05).

As shown in Figure 7A,B (mean α_S1_- and α_S2_-caseins concentration between 6–20 °C at 0–7.5 mM added calcium), the concentration of α_S_-caseins decreases with increasing calcium concentration. Small amounts of added calcium (0.25 mM) are sufficient to affect the α_S_-caseins due to their high calcium affinity [24,29]. The serum concentration of α_S2_–casein decreases from 0.4 g L^−1^ at native conditions to 0.28 g L^−1^ upon addition of 0.25–0.5 mM calcium and of α_S1_–casein to 0.21 g L^−1^ with 5–7.5 mM added calcium. The serum concentration of α_S1_–casein as a function of added calcium is comparable to the one of α_S2_ but at a lower level. The observed effect is independent of the temperature. Therefore, it can be summarized that besides β-casein also α_S_‑caseins are decreased by addition of calcium. However, α_S2_–casein is only decreased to a certain concentration of approx. 0.24 g L^−1^ which cannot be decreased further by adding more calcium than 0.25 mM.

**Figure 8:**
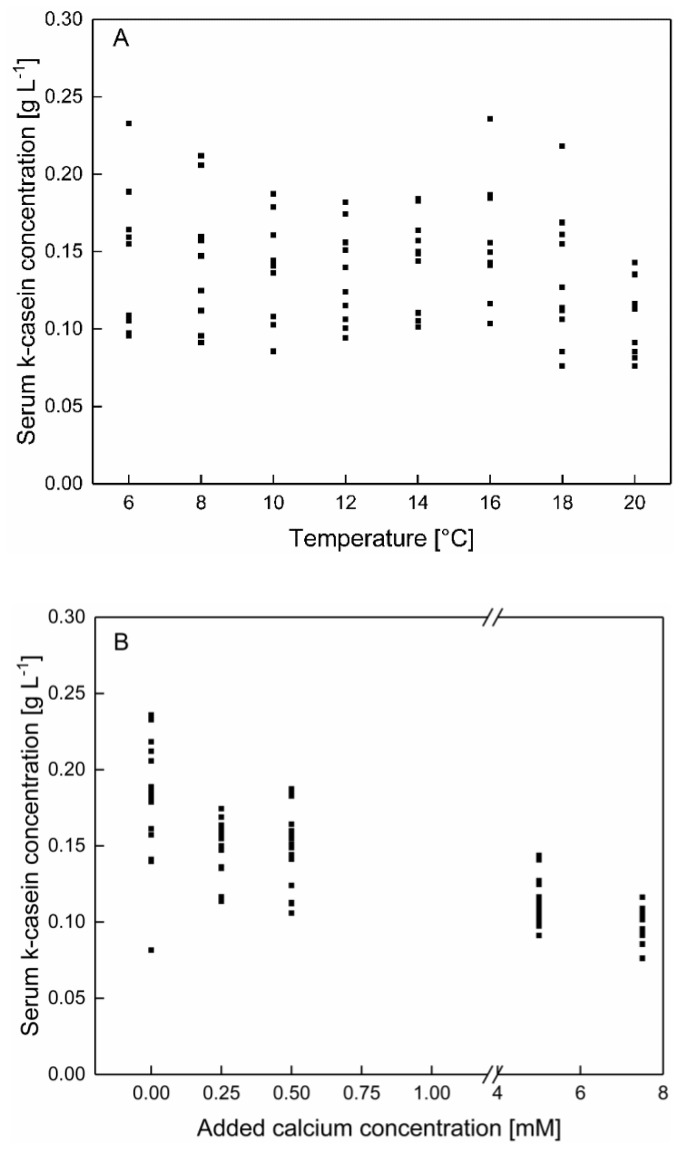
κ-casein concentration in the serum phase of skim milk (**A**) as a function of the temperature with an addition of 0–7.5 mM calcium and (**B**) as a function of added calcium at 6–20 °C (*n* = 2). Data points are individual experiments. κ-casein concentration (between 6–20 °C) decreased significantly with increasing calcium addition (*p* < 0.05).

In contrast to the data shown in Section 3.1 κ-casein shows after the enrichment of skim milk with calcium a significant dependency as a function of calcium addition as well as of temperature (*p* < 0.05), even though at native conditions no temperature dependency could be observed. The κ-casein concentrations in Figure 8A is shown as a function of the temperature, regardless of the calcium concentration and in in Figure 8B as a function of the calcium concentration, regardless of the temperature. Therefore, an impact of temperature and calcium concentration can be separated from one and another.

As shown in Figure 8A no specific shift of the κ-casein concentrations as a function of the temperature can be observed.

Regarding the calcium enrichment, a decrease of the serum κ-casein concentration with increasing calcium concentration can be observed (Figure 8B). The addition of calcium can increase the affinity for κ-casein to bind to micellar structures as well as to casein aggregates, likely due to the decrease of the pH following the calcium enrichment. This can may be attributed to a change in the zeta potential of the micellar surface following changes in the composition and protonation of ions and proteins at the micellar surface [30].

In summary, the addition of calcium can decrease the absolute serum casein concertation at low temperatures and counterbalance the temperature dependency of the serum casein concentration. At low temperatures, predominantly β-casein is affected, because of the high percentage in serum casein composition. Furthermore, regardless of the affinity to calcium ions the concentration of the other casein species in the serum decreases after addition of calcium.

### 3.5. Decreasing Phosphate and Citrate Concentration after the Addition of Calcium

To understand the effect of calcium on the casein equilibrium, the determination of anions as a function of added calcium can be studied. Added calcium can bind either to casein aggregates or with anions to form inorganic complexes.

The serum concentration of soluble phosphate and citrate as a function of added calcium is shown in Table 2. The concentration of both cations was constant between 0.00–0.50 mM of added calcium, whereas the total serum casein concentration decreases significantly after the addition of 0.50 mM calcium (Figure 5) (*p* < 0.05). The α_S_-caseins decrease after the addition of 0.25 mM calcium (Figure 7). Therefore, the added calcium forms ion complexes with the soluble caseins with a higher affinity than it is bound to soluble cations. After the addition of 5 mM calcium, the concentration of phosphate and citrate decreases in the soluble phase, due to the formation of inorganic ion complexes [30,31,32]. A further increase of the added calcium concentration decreases the soluble anion concentration. Therefore, a precise determination of the calcium interaction with calcium sensitive casein species can be seen after the addition of low amounts of calcium, whereas the reduction of the cations occurs after a certain amount of calcium is exceeded.

### 3.6. Influence of Calcium Concentration on the Serum Casein Concentration with pH Adjustment

**Figure 9:**
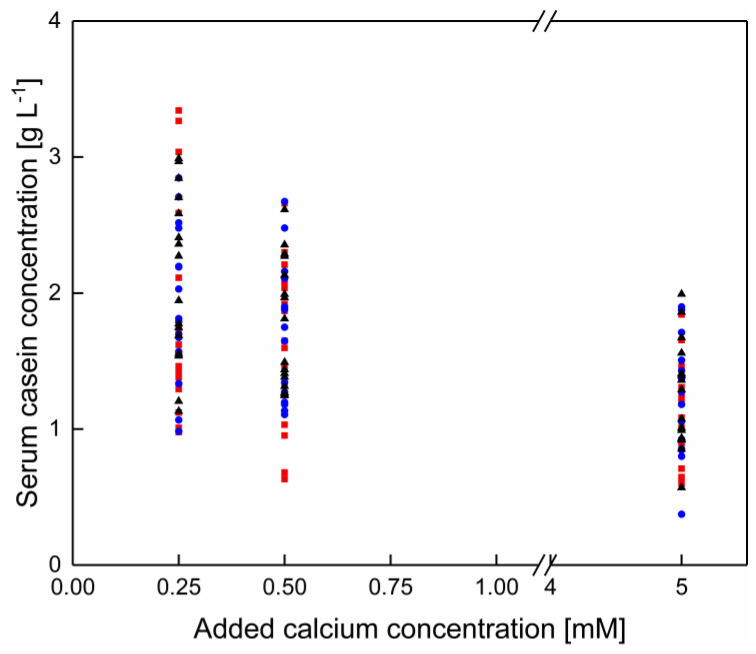
Total casein concentration in the serum phase of skim milk at 6–20 °C after the addition of 0–5.0 mM without pH adjustment (■) and with pH adjustment to pH 6.8 (●) and 7.3 (▲) after calcium addition (*n* = 2). Data points are individual experiments. No significant difference of the serum casein concentration after calcium addition as a function of the pH adjustment (*p* > 0.05).

To assess whether there was an effect of the pH on the serum casein concertation at lower temperatures as a result of a calcium enrichment, the pH was adjusted to 6.8 and 7.3 after the addition of 0.25, 0.50, and 5.0 mM of calcium. The serum casein concentration is shown in Figure 9 as a function of the added calcium concentration without as well as with a pH adjustment to 6.8 and 7.3, independently of the temperature. It can be seen that independently of the pH the casein concentration decreases in the serum phase after the addition of up to 0.50 mM calcium. Therefore, regardless of whether a pH adjustment after calcium addition was performed, the serum casein concentration was reduced with increasing calcium concentration. Concluding, the pH reduction following the addition of calcium has no significant effect on the total serum casein concentration (*p* > 0.05). This observation is in accordance with the data shown by Philippe et al. [30]. The authors showed that the re-adjustment of the pH to 6.75 after the addition of 0–13.5 mM calcium did not affect the protein concentration in the supernatant, but only at room temperature and not for temperatures as low as 6 °C.

Nevertheless, the formation or dissolution of different ion complexes depends on the amount of calcium addition and on the pH adjustment [32]. Therefore, after the addition of calcium and adjustment of the pH, only the concentration of hydrogen ions and thus, the electrostatic repulsion is similar in the serum phase. However, the composition of the ion complexes and their accessibility by the caseins is likely to be different depending on the mode of adjustment, i.e., the composition of the added ions and their interaction with the native milieu.

In contrast to the total serum casein, the α_S2_-casein concentration is affected by the pH adjustment after the addition of calcium. An increasing concentration can be observed, when the pH is adjusted after an addition of 0.50 or 5.00 mM calcium to 6.8 or 7.3, independently of the temperature (Table 3). This effect occurs even though no significant effect of the pH was noticeable when it was adjusted without a calcium addition (*p* > 0.05). Furthermore, in Section 3.4 a reduction of the α_S2_ serum casein concentration was shown with an enhanced calcium concentration of 0.50 and 5.00 mM calcium. Therefore, the increased ionic strength in combination with an increased pH reduces the electrostatic repulsion of the α_S2_-casein increasing the amount in the soluble fraction. Regarding α_S1_-, β-, and κ-casein no significant effect of a pH adjustment after calcium addition could be observed (data not shown) (*p* > 0.05). Therefore, for those species, the change in pH resulting from the calcium enrichment does not influence the concentration in the serum phase. 

### 3.7. Comparison of the Serum Casein Concentration after Ultracentrifugal Separation with the Concentration in Microfiltration Permeate

As shown by Schiffer et al. [17] the casein permeation can be reduced in MF permeate by the addition of 5 mM calcium to skim milk at 10 and 14 °C. However, after the addition of 5 mM calcium also a decrease in the whey protein permeation occurred. To determine whether the reduced casein permeation was following a decreasing serum casein concentration prior to the filtration or an alteration of the deposit layer during the MF, which also decreased the whey protein permeation, the results were compared with the ones in this study.

It can be seen in Table 4 that the serum casein concentration in MF permeate is significantly lower compared to the concentration in the ultracentrifugal supernatant (*p* < 0.05). Therefore, the deposited layer in the microfiltration experiment leads to a high casein retention, with and without addition of calcium. However, for both sets of experiments, a decreased serum casein concentration can be determined after the addition of 5 mM calcium. A comparison of the casein concentration in the MF permeate with the serum casein concentration in the supernatant after ultracentrifugation, enables the determination of the retention of soluble casein by the deposited layer of retained casein micelles during microfiltration. Thus, it can be determined if an alteration of the deposit layer leads to the reduced serum casein permeation (Table 4).

At native conditions without calcium addition, a casein permeation relative to the milk serum of 27% at 10 °C and 29% at 14 °C, respectively, was observed. After the addition of 5 mM calcium the permeation of serum casein was decreased to 24% at 10 and 25% at 14 °C, respectively. Therefore, the predominant factor on the reduced casein permeation during microfiltration with an enhanced calcium concentration was the reduced serum casein concentration. An alteration of the deposit layer has only a minor effect on the reduced casein permeation, since the main effect of calcium addition in this regard already occurs in the serum prior to microfiltration by shifting the casein equilibrium towards the casein micelles. However, even though the casein permeation is reduced due to a binding of soluble caseins prior to the filtration process, the whey protein permeation is distinctly affected as a result of the changes in the deposit layer resulting from the calcium addition. 

These results mean that an optimization of the microfiltration process would be possible by an addition of calcium, provided that the unintended effect on a tighter structure of the deposit casein micelle layer and its retention capacity for whey proteins would be minimized. For instance, a minimal deposit formation would be achieved if the length dependence of deposit formation would be avoided [33], e.g., by applying the so-called UTP-concept (uniform transmembrane pressure) or gradient membranes [12,34].

## 4. Conclusions

The concentration and composition of caseins in milk serum varied as a function of the temperature between 6–20 °C. It was shown that a considerable improvement of the fractionation of caseins and whey proteins by microfiltration process modifications with regard to the temperature, pH, and/or ion addition can be achieved. 

A calcium addition to skim milk prior to microfiltration can decrease the total serum casein concentration. Thus, an increased β-casein content in the milk serum at lower temperature can be prevented. A reduction of the temperature as low as 6 °C can be considered for the MF of skim milk after the addition of 5.00 mM calcium without increasing the casein concentration in the serum phase compared to 20 °C. However, this level of calcium addition would also tighten the deposited casein micelle layer structure and affect the desired high permeation of whey proteins into the MF permeate.

Furthermore, the addition of calcium chloride to decrease the total serum casein concentration affects all casein species at different extents, changing the composition of the casein equilibrium in the aqueous phase. Thus, even though the total serum casein concentration in skim milk at high temperatures without calcium enrichment, is similar to the one at low temperatures with calcium enrichment, the composition is different. Therefore, the composition of the casein fraction reducing the purity of the whey protein fraction can be controlled by the addition of calcium. A more effective deposit layer control as in conventional crossflow filtration would, however, be a condition if the application of higher levels of calcium addition would be considered.

## Figures and Tables

**Table 1:** Mean values of calcium, phosphate citrate and magnesium concentrations between 6–20 °C in the serum phase of skim milk at pH 6.3–7.3.^a.^

	Calcium [mg L^−1^]	Phosphate [mg L^−1^]	Citrate [mg L^−1^]	Magnesium [mg L^−1^]
pH 6.3	460 ± 40	1316 ± 45	1768 ± 116	83 ± 5
pH 6.5	400 ± 20	1189 ± 45	1479 ± 173	76 ± 4
pH 7.0	315 ± 14	1090 ± 30	1500 ± 155	78 ± 4
pH 7.3	314 ± 7	986 ± 27	1671 ± 59	75 ± 4

^a^ The ion concentration changed not significantly as a function of the temperature (6–20 °C) (*p* > 0.05). Calcium, phosphate and citrate changed significantly as a function of a pH increase from pH 6.3–7.3 (*p* < 0.05).

**Table 2:** Mean values of phosphate and citrate concentration of skim milk between 6–20 °C at different levels of calcium addition ^a^.

	Phosphate [mg L^−1^]	Citrate [mg L^−1^]
0.00 mM Ca^2+^	1029 ± 39	1557 ± 81
0.25 mM Ca^2+^	1014 ± 49	1541 ± 76
0.50 mM Ca^2+^	1068 ± 34	1597 ± 62
5.00 mM Ca^2+^	1013 ± 31	1427 ± 21
7.50 mM Ca^2+^	951 ± 26	1203 ± 65

^a^ The phosphate and citrate concentration do not change significantly as a function of the temperature (6–20 °C) (*p* > 0.05). Changes as a function of a calcium increase from 0.00–7.5 mM are significant (*p* < 0.05).

**Table 3:** Mean α_S2_-casein concentration in the serum phase of skim milk between 6–20 °C after addition of 0.5 mM and 5 mM calcium without and with pH adjustment to 6.8 and 7.3, respectively. ^a.^

	0.5 mM Added Calcium	5 mM Added Calcium
Without pH adjustment	0.27 ± 0.05	0.22 ± 0.04
pH adjustment to 6.8	0.51 ± 0.02	0.47 ± 0.06
pH adjustment to 7.3	0.51 ± 0.02	0.46 ± 0.05

^a^ Significant difference of the serum casein concentration after calcium addition between no pH adjustment and a pH adjustment to 6.8 or 7.3 (*p* < 0.05).

**Table 4:** Comparison of serum casein and whey protein concentration in microfiltration (MF) permeate as shown by Schiffer et al. [17] with serum casein concentration in ultracentrifugal supernatant at 10 and 14 °C without and with 5 mM added calcium.

		Native		5 mM Added Calcium	
		10 [°C]	14 [°C]	10 [°C]	14 [°C]
Serum casein conc. [g L^−1^]	MF permeate	0.83	0.70	0.37	0.35
	Supernatant	3.05	2.43	1.57	1.4
Relative serum casein permeation [%]		27	29	24	25
Whey protein conc. in MF permeate [g L^−1^]		1.9	2.1	1.3	1.4

## Appendix A

**Table A1:** pH of skim milk as a function of the temperature between 6–20 °C.

	6 °C	8 °C	10 °C	12 °C	14 °C	16 °C	18 °C	20 °C
pH [-]	6.78	6.77	6.77	6.75	6.75	6.75	6.73	6.72

**Table A2:** Mean value of the anion concentration in the serum phase of skim milk between 6–20 °C.

Citrate [mg L^−1^]	Phosphate [mg L^−1^]	Chloride [mg L^−1^]
1557 ± 81	1030 ± 39	838 ± 37

**Table A3:** Mean value of the cation concentration in the serum phase of skim milk between 6–20 °C.

Potassium [mg L^−1^]	Sodium [mg L^−1^]	Calcium [mg L^−1^]	Magnesium [mg L^−1^]
1562 ± 70	391 ± 15	320 ± 31	79 ± 7
